# Origin of giant piezoelectric effect in lead-free K_1−*x*_Na_*x*_Ta_1−*y*_Nb_*y*_O_3_ single crystals

**DOI:** 10.1038/srep25637

**Published:** 2016-05-10

**Authors:** Hao Tian, Xiangda Meng, Chengpeng Hu, Peng Tan, Xilong Cao, Guang Shi, Zhongxiang Zhou, Rui Zhang

**Affiliations:** 1 Department of Physics, Harbin Institute of Technology, Harbin 150001, China; 2 Condensed Matter Science and Technology Institute, Harbin Institute of Technology, Harbin 150001, China

## Abstract

A series of high-quality, large-sized (maximum size of 16 × 16 × 32 mm^3^) K_1−*x*_Na_*x*_Ta_1−*y*_Nb_*y*_O_3_ (*x* = 0.61, 0.64, and 0.70 and corresponding *y* = 0.58, 0.60, and 0.63) single crystals were grown using the top-seed solution growth method. The segregation of the crystals, which allowed for precise control of the individual components of the crystals during growth, was investigated. The obtained crystals exhibited excellent properties without being annealed, including a low dielectric loss (0.006), a saturated hysteresis loop, a giant piezoelectric coefficient *d*_33_ (*d*_33_ = 416 pC/N, determined by the resonance method and *d*_33_^*^ = 480 pC/N, measured using a piezo-*d*_33_ meter), and a large electromechanical coupling factor, *k*_33_ (*k*_33_ = 83.6%), which was comparable to that of lead zirconate titanate. The reason the piezoelectric coefficient *d*_33_ of K_0.39_Na_0.61_Ta_0.42_Nb_0.58_O_3_ was larger than those of the other two crystals grown was elucidated through first-principles calculations. The obtained results indicated that K_1−*x*_Na_*x*_Ta_1−*y*_Nb_*y*_O_3_ crystals can be used as a high-quality, lead-free piezoelectric material.

Piezoelectric materials are used widely in a variety of applications, including in ultrasonic transducers and piezoelectric transformers and sensor. For the last 60 years, lead zirconate titanate (PZT) ceramics were the most popular piezoelectric materials, owing to their excellent piezoelectric properties^1^,^2^,^3^. However, because lead causes significant harm to the environment and human health, efforts have been made to develop lead-free piezoelectric materials^4^,^5^. It is widely believed that potassium sodium niobate (K_1−x_Na_x_NbO_3_, KNN)-based materials have great potential as replacements for lead-based piezoelectric materials because of their excellent properties and because their Curie temperatures (*T*_C_) are higher than those of other lead-free materials such as barium titanate (BT) and bismuth sodium titanate (BNT)^6^,^7^,^8^.

In 2004, Y. Saito *et al*. reported the large piezoelectric effect in Li, Ta, Sb co-doped KNN ceramics. A major breakthrough was the fact that the piezoelectric coefficient *d*_33_ of these KNN-based ceramics was as high as 416 pC/N^6^. Since then, intensive research efforts have been made to improve the piezoelectric properties of KNN-based ceramics^9^,^10^,^11^,^12^,^13^,^14^,^15^,^16^. As is known, the properties of single crystals are better than those of ceramics of the same composition. Furthermore, in order to investigate the mechanism responsible for the large piezoelectric effect observed in doped KNN and to improve the performance of KNN-based materials, it is important to grow KNN-based single crystals. Recently, several methods for growing KNN-based crystals have been reported, and each method has its advantages and disadvantages. The most widely used methods for growing KNN-based single crystals are the flux, Bridgman, and top-seed solution growth (TSSG) methods. The flux method yields crystals of good quality; however, the as-grown crystals are difficult to study, owing to their small size^17^,^18^,^19^. Chen *et al*. successfully grew Li:KNN single crystals with a large *d*_33_ (405 pC/N) using the Bridgman method; however, the single crystals contained oxygen vacancies and defects, which led to a high leakage current, as was evident from their polarization vs. electric field hysteresis loops^20^. Zheng *et al*. grew Li, Ta, Sb-doped KNN single crystals using the TSSG method and investigated their piezoelectric properties. They were able to obtain K_0.561_Na_0.439_Nb_0.768_ Ta_0.232_O_3_ (*d*_33_ = 200 pC/N, *k*_33_ = 82.7%), (K_0.487_Na_0.513_Li_0.01~0.04_) (Nb_0.667_ Ta_0.333_) O_3_ (*d*_33_ = 354 pC/N, *k*_33_ = 81.5%), and (K_0.51_Na_0.49_Li_0.02~0.03_)(Nb_0.67_Ta_0.32_ Sb_0.01_)O_3_ (*d*_33_ = 172 pC/N, *k*_33_ = 52.3%)^21^,^22^,^23^. For the lack of continuous changes in the composition and doping ions, the research of the contribution of doping ions on the piezoelectric properties of KNN is difficult. Furthermore, there have been few microscopic studies on the physical mechanism responsible for the improvement in the piezoelectric properties after doping with Ta.

In this paper, we report the successful growth of a series of high-quality, large-sized K_1−x_Na_x_Ta_1−y_Nb_y_O_3_ (KNTN) single crystals using the TSSG method. The segregation of the crystals, which allowed for precise control over the individual components of the crystals, was investigated. The as-grown crystals exhibited excellent properties, including a saturated hysteresis loop, a giant piezoelectric coefficient, *d*_33_^*^ (480 pC/N), and a large electromechanical coupling factor, *k*_33_ (83.6%); the values of these parameters were comparable to those of PZT. Finally, the origin of the high piezoelectric effect observed in the crystals was elucidated through first-principles calculations.

## Results and Discussion

**Figure 1:**
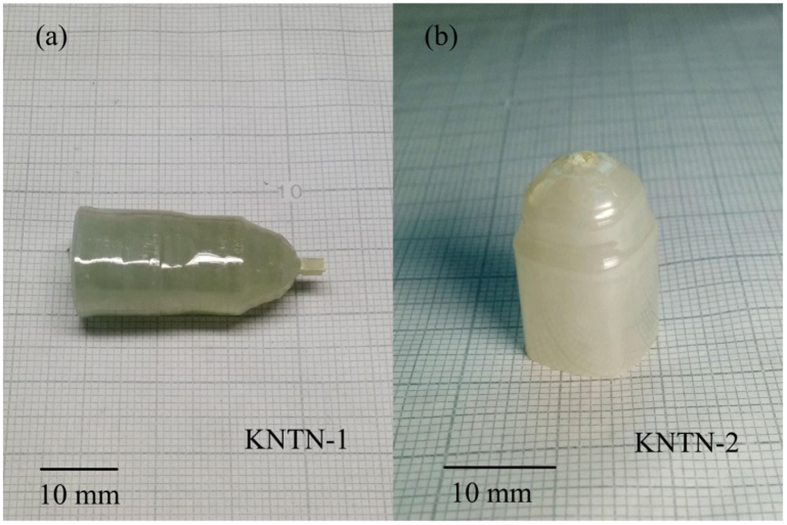
Photographs of the as-grown KNTN single crystals.

Photographs of the as-grown KNTN crystals are shown in Fig. 1. The dimensions of the largest KNTN crystal grown were 16 × 16 × 32 mm^3^, which are much greater than those of previously reported KNTN crystals. The crystals were transparent at temperatures higher than their Curie temperature (*T*_C_), and no cracks were observed in them. At room temperature, the crystals were milky white, because of the presence of polydomains, which scatter light. The KNTN crystals were shaped like a square with round corners, and the surfaces of the crystals were (100)_C_ and (010)_C_ faces.

The segregation of the crystals allowed for precise control of the individual components of the crystals during the growth process. The relationships between the composition of the KNTN crystals; the ratio of the potassium and sodium concentrations in the melt; the ratio of the tantalum and niobium concentrations in the melt; the segregation coefficient of potassium, sodium, tantalum, and niobium; and the growth temperature (*T*_g_) are shown in Table 1. The segregation coefficient of potassium (*S*^K^) was calculated from the equation: *S*^K^ = *C*_*c*_^K^*/C*_*m*_^K^, where *C*_*c*_^K^ is K concentration in single crystal; *C*_*m*_^K^ is K concentration in melt. The similar calculations were performed for the *S*^Na^, *S*^Ta^, *S*^Nb^. The segregation of ion was determined by their properties. Moreover, it was also determined by ion concentration in melt and the condition of growth. With an increase in the tantalum content in the melt, the growth temperature increased from 1193 °C to 1277.5 °C. On the other hand, the segregation coefficient of tantalum decreased from 3.700 to 2.333, which in accordance with the phase diagram of the KTaO_3_-KNbO_3_ system^24^. Further, the segregation coefficient of potassium increased from 0.400 to 0.520, while that of sodium decreased from 2.800 to 2.560. This was in contradiction to the phase diagram of the KNbO_3_-NaNbO_3_ system^25^ and meant that the doping of Ta into the KNN system affected the segregation of K and Na.

**Figure 2:**
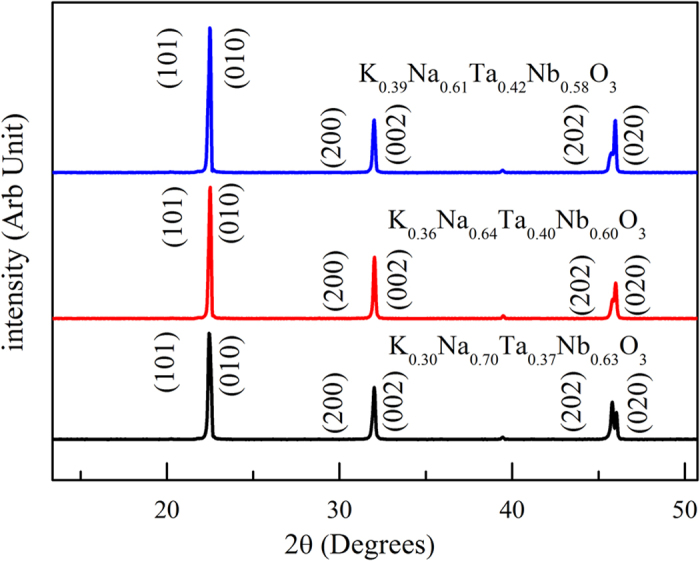
X-ray powder diffraction patterns of the as-grown K_1−x_Na_x_Ta_1−y_Nb_y_O_3_ crystals.

The XRD patterns indicated that the crystals had pure perovskite-like structures and did not contain any secondary phases (Fig. 2). The as-grown KNTN single crystals were in the orthorhombic phase at room temperature. From the XRD data, the lattice parameters of the KNTN crystals were determined using the software Jade 6.0; the results are shown in Table 2. The (202) and (020) peaks moved to the high degrees decreases in x and y, resulting in increases in the lattice parameters “a” and “b.” These changes in the lattice parameters of the KNTN crystals indicated that the volume of the lattice cell increased with the decrease in x and y. The cation radius of the tantalum *r*(Ta^5+^) is slightly smaller than that of the niobium *r*(Nb^5+^), while the volume of the as-grown crystals increase with decrease of Nb concentration (as Table 2 shown). Thus the main reason for the increase in the volume was the fact that the cation radius of potassium *r*(K^+^) is larger than that of sodium *r*(Na^+^).

**Figure 3:**
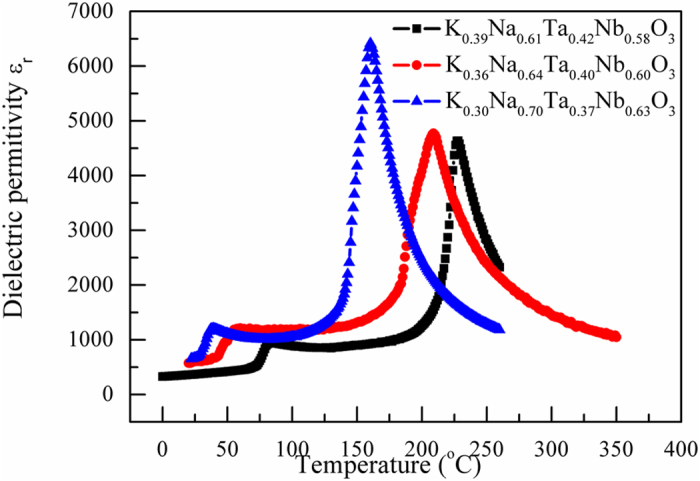
Temperature dependence of the dielectric constant of the K_1−x_Na_x_Ta_1−y_Nb_y_O_3_ crystals at 1 kHz.

The temperature dependence of the dielectric constant of the K_1−x_Na_x_Ta_1−y_Nb_y_O_3_ crystals at 1 kHz is shown in Fig. 3. The two peaks in the curves represent the orthorhombic-tetragonal phase-transition temperature (*T*_O-T_) and the Curie temperature (*T*_C_), respectively; the values of these temperatures were consistent with the XRD results and suggested that the crystals were in the orthorhombic phase at room temperature. As can be seen from the data listed in Table 3, with the tantalum and sodium content varying, the value of *T*_O-T_ of the KNTN crystals decreased from 84 °C to 39 °C. Concomitantly, the value of *T*_C_ decreased from 228 °C to 161 °C. According to the data of pure KNN single crystals^26^, we can determine that the value of *T*_O-T_ and *T*_C_ of the KNN crystals slightly increased with decrease of Na concentration. However, the *T*_C_ of KTN single crystals gotten by the function *T*_C_ = 676*x* + 32 K (*x* is the Nb concentration in KTN crystals)^27^ decreased with doping Ta. Thus, the variation of *T*_O-T_ and *T*_C_ mainly owed to Ta/Nb ratio^28^. In addition, the dielectric property (*ε*_*r*_) of the as-grown KNTN crystals increased with the increase in the potassium and tantalum contents, and is larger than that of the pure KNN single crystals. The result is mainly reasonable because Ta could move the *T*_O-T_ peak left, and room temperature is close to the *T*_O-T_ peak. The quality of K_0.39_Na_0.61_Ta_0.42_Nb_0.58_O_3_ was better than the other two components crystals as its tan*δ* was the smallest. Furthermore, it was similar or smaller than that of the crystals reported previously in the literature^26^,^29^.

**Figure 4:**
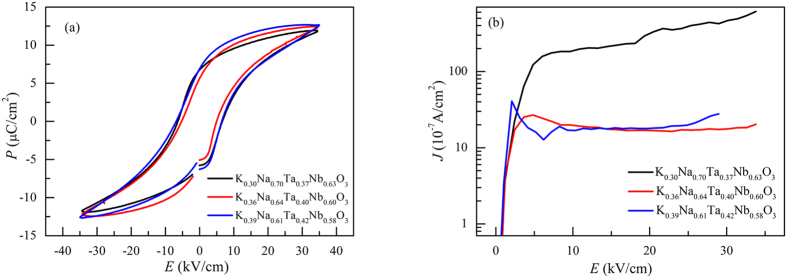
(**a**) *P-E* hysteresis loops of the KNTN crystals at room temperature at a frequency of 200 Hz. (**b**) Values of the leakage current density (*J*) at room temperature along the [001]_C_ direction of the KNTN crystals.

Figure 4a showed the *P-E* hysteresis loops of the KNTN crystals at 25 °C at a frequency of 200 Hz under a maximum electric field of 35 kV/cm. All as-grown crystals exhibit saturated curves at an electric field of 35 kV/mm, which suggests that as-grown crystals were of high quality. The values of the coercive field (*E*_c_), remanent polarization (*P*_r_), and spontaneous polarization (*P*_s_) of the KNTN single crystals as well as those of the pure KNN single crystals are listed in Table 4. The *E*_c_, *P*_r_, and *P*_s_ values of the KNTN crystals were smaller than those of pure KNN single crystals. The result is in good agreement with the difference between Ta^5+^ and Nb^5+^ in unit cell. Ta^5+^ and Nb^5+^ ions were randomly distributed in the B-sites. Because Nb^5+^ ions moving along the 12 directions of spontaneous polarization mainly contribute to the *P*_r_, the *P*_r_ decreased with doping Ta. The values of the room-temperature leakage current density (*J*) along the [001]_C_ direction of the as-grown KNTN crystals are shown in Fig. 4b. The leakage currents for the KNTN crystals which were not annealed at high temperature had an order of magnitude of ~10^−5^ A/cm^2^, which is far lower than that for KNN crystals grown by the flux method (10^−3^ to 10^−4^ A/cm^2^)^30^,^31^. This result suggested that there were less O^2−^ vacancies in the crystals grown by TSSG method^26^, which was another indication of the high quality of the KNTN single crystals grown in this study. Because of the more O^2−^ vacancies of K_0.30_Na_0.70_Ta_0.37_Nb_0.63_O_3_ caused by the lower melting temperature, the *J* of that was one order of magnitude larger than other two single crystals.

**Figure 5:**
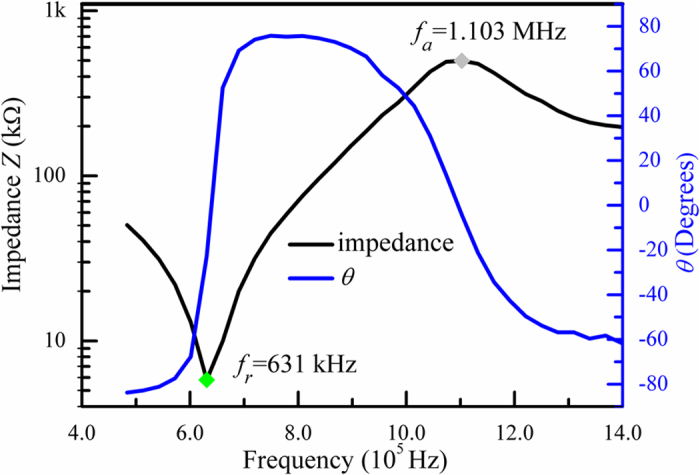
Room-temperature *k*_33_ resonator impedance spectra of K_0.39_Na_0.61_Ta_0.42_Nb_0.58_O_3_ single crystal.

The piezoelectric vibrators were cut along the [001]_c_ direction, and their impedance spectra (magnitude of impedance |*Z*| and phase angle, *θ*, vs. frequency) were measured, so that their piezoelectric parameters could be calculated. A typical longitudinal extension response for the *k*_33_ resonator of K_0.39_Na_0.61_Ta_0.42_Nb_0.58_O_3_ single crystal was shown in Fig. 5. For this *k*_33_ resonator, a resonance frequency (*f*_*r*_) was 631 kHz while an antiresonance frequency (*f*_*a*_) was 1.103 MHz. The values of the elastic compliance constant *s*_33_, electromechancical coupling factor *k*_33_, and piezoelectric coefficient *d*_33_ of the samples were calculated by substituting the measured long-size *l, f*_*r*_ and *f*_*a*_ into the following equation: 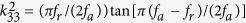, 
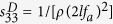
, 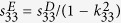, 
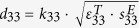
. The maximum phase angle was approximately 75°; this was indicative of the existence of a polydomain state (domain engineered). The similar measurement was performed for other *k*_33_, *k*_31_, and *k*_t_ resonators. The *k*_33_ values of the samples were between 76.4% and 83.6% and larger than those reported previously for KNN-TL and PZT5A. Further, the giant piezoelectric coefficient *d*_33_ of the K_0.39_Na_0.61_Ta_0.42_Nb_0.58_O_3_ crystal was 416 pC/N, as determined by the resonance method, while *d*_33_^*^ was 480 pC/N, as measured with a piezo-*d*_33_ meter; these values are larger than those for other two as-grown single crystals and KNN-TL. As can be seen from the data, the values of most of the parameters of K_0.39_Na_0.61_Ta_0.42_Nb_0.58_O_3_ which were greatly influenced by temperature were comparable to those of PZT5A.

As shown in Table 5, the piezoelectric coefficient *d*_33_ of the K_0.39_Na_0.61_Ta_0.42_Nb_0.58_O_3_ crystal was larger than those of the K_0.30_Na_0.70_Ta_0.37_Nb_0.63_O_3_ and K_0.36_Na_0.64_Ta_0.40_Nb_0.60_O_3_ crystals. This can be attributed to the polymorphic phase transition (PPT) which was lowered to room temperature by Ta, when the K/Na ratio was far away from 0.5/0.5^32^,^33^. However, the microscopic physical mechanism responsible was not involved. To elucidate the origin of the high piezoelectric effect in the microcosmic, we used first-principles calculations to calculate the free energy of the lattice cell.

**Figure 6:**
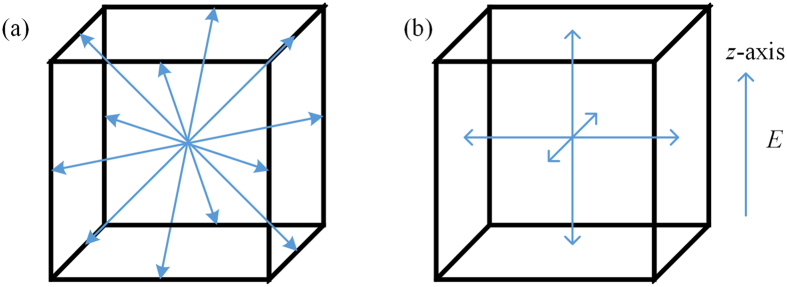
Directions of spontaneous polarization of the KNTN crystals in the orthorhombic and tetragonal phases: (**a**) spontaneous polarization directions of the orthorhombic phase and (**b**) spontaneous polarization directions of the tetragonal phase.

Spontaneous polarization occurs in twelve directions along [011] _C_ in the orthorhombic (*mm*2)-phase single crystals, while it occurs in six directions along [001]_C_ for the tetragonal (4 *mm*) -phase single crystals (shown in Fig. 6). When an electric field was applied along the *z*-axis, the directions in which spontaneous polarization occurred in the orthorhombic phase changed to those corresponding to the tetragonal phase. In addition, according to a previous study, the reason the piezoelectric coefficient *d*_33_ of the grown crystals was large was that the polarization directions were rotated and not stretched^34^. The internal energies of the K_1−x_Na_x_Ta_1−y_Nb_y_O_3_ crystals corresponding to the different phases (orthorhombic phase*, U*_o_, and tetragonal phase, *U*_t_) were calculated through first-principles calculations. For this, we calculated the internal energies of potassium tantalate (KTaO_3_, KT), potassium niobate (KNbO_3_, KN), sodium tantalate (NaTaO_3_, NT), and sodium niobate (NaNbO_3_, NN), as well as the weighted averages^35^,^36^ of the individual coefficients. The obtained results are shown in Table 6.

It can be seen that Δ*U* decreased from 3.0241 eV/Å^3^ to 2.5666 eV/Å^3^ with an increase in the Ta fraction from 0.37 to 0.42 and an increase in the K fraction from 0.30 to 0.39. Further, the difference in the free energies of the orthorhombic and tetragonal phases of K_0.39_Na_0.61_Ta_0.42_Nb_0.58_O_3_ in the absence of an electric field was the lowest. Thus, when an electric field was applied, the domain of K_0.39_Na_0.61_Ta_0.42_Nb_0.58_O_3_ which was at PPT temperature, was the easiest to rotate, which can enhance piezoelectric properties^34^. The conclusion was the same as that calculated by Landau – Devonshire model in BaTiO_3_ – based materials^37^,^38^ and PZT-based materials^39^. As a result, its piezoelectric coefficient was larger than those of K_0.30_Na_0.70_Ta_0.37_Nb_0.63_ O_3_ and K_0.36_Na_0.64_Ta_0.40_Nb_0.60_O_3_.

## Conclusions

In this study, a series of large-sized (size of largest crystal = 16 × 16 × 32 mm^3^) orthorhombic K_1−x_Na_x_Ta_1−y_Nb_y_O_3_ (x = 0.61, 0.64, and 0.70 and corresponding y = 0.58, 0.60, and 0.63) single crystals were grown using the TSSG method. The crystals exhibited excellent dielectric, piezoelectric, and ferroelectric properties, including a giant *d*_33_^*^ (480 pC/N) and a large *k*_33_ (83.6%). The leakage currents of the K_1−x_Na_x_Ta_1−y_Nb_y_O_3_ crystals were very low and of the order of 10^−5^ A/cm^2^. The piezoelectric properties of the K_1−x_Na_x_Ta_1−y_Nb_y_O_3_ crystals improved with an increase in the potassium and tantalum contents when the phase of the K_1−x_Na_x_Ta_1−y_Nb_y_O_3_ crystals was orthorhombic. Finally, the difference in the free energies of the orthorhombic and tetragonal phases of K_0.39_Na_0.61_Ta_0.42_Nb_0.58_O_3_ in the absence of an electric field was 2.5666 eV/Å^3^; this was determined by first-principles calculations. This crystal was the smallest of the grown single crystals. Further, its domain was the easiest to rotate. Therefore, it exhibited the best piezoelectric properties.

## Methods

As mentioned above, the KNTN single crystals were grown by the TSSG method. The raw materials used were powders of K_2_CO_3_ (99.99%), Na_2_CO_3_ (99.99%), Ta_2_O_5_ (99.99%), and Nb_2_O_5_ (99.99%). They were weighted to obtain a composition of K_1−*x*_Na_*x*_Ta_1−*y*_Nb_*y*_O_3_, with 10 mol% excess of K_2_CO_3_ and Na_2_CO_3_ ((K_2_CO_3_ + Na_2_CO_3_):(Ta_2_O_5_ + Nb_2_O_5_) = 1.1:1) as the self-flux. The raw materials were mixed with ethanol, ball-milled for 24 h, and subsequently dried in an oven at 85 °C to volatilize the ethanol. Then, the mixture was calcined at 950 °C for 6 h to synthesize a KNTN polycrystal. The polycrystal was melted in a medium-frequency induction furnace at 1250 °C ~ 1375 °C, which is ~100 °C higher than the temperature for crystal growth, in order to eliminate the residual carbon dioxide and mix the compounds at the atomic level. Then, the temperature was decreased to the growth temperature, and a single crystal began to grow on a [001]_C_ seed that was cut from a high-quality potassium tantalate niobate crystal. During crystal growth, the rotational and pulling rates were 15 r/min and 0.25 mm/h, respectively. After the completion of the growth process, the as-grown crystal was cooled to the room temperature at 35 °C/h.

The compositions of the as-grown crystals were determined by electron microprobe analysis (EPMA-1720, Shimadzu, Kyoto, Japan). The structures of the crystals were confirmed by X-ray diffraction (XRD) analyses (XRD-6000, Shimadzu, Kyoto, Japan). The crystals were oriented using a Laue X-ray machine. The (001)_C_ in pseudo cubic structure structure surfaces of the samples were covered with silver electrodes, and the dielectric properties of the samples were measured as functions of the temperature using an inductance – capacitance − resistance (LCR) meter (E4980A, Agilent Technologies, Santa Rosa, CA). The polarization vs. electric field (*P-E*) hysteresis loops of the crystals were measured at 200 Hz using the ferroelectric test system (Precision Premier II, Radiant Technology, Inc., Albuquerque, NM, USA); the leakage current densities of the crystals were recorded using the same instrument. Cuts of the crystals with dimensions similar to those mentioned in IEEE standards were poled in silicon oil at a temperature of *T*_O-T_ −10 °C under an electric field of 30 kV/cm. The resonance and antiresonance frequencies were measured using an HP 4294 A impedance phase analyzer. The piezoelectric coefficients and electromechanical coupling factors were determined at the resonance and antiresonance frequencies, according to the IEEE standards. The piezoelectric constant *d*_33_^*^ was measured using a piezo-*d*_33_ meter (Zj-3A, Institute of Acoustics, Academic Sinica, Beijing, China).

## Additional Information

**How to cite this article**: Tian, H. *et al*. Origin of giant piezoelectric effect in lead-free K_1−*x*_ Na_*x*_Ta_1−*y*_ Nb_*y*_O_3_ single crystals. *Sci. Rep.* **6**, 25637; doi: 10.1038/srep25637 (2016).

## Figures and Tables

**Table 1:** Segregation of potassium, sodium, tantalum, and niobium and the growth temperatures of K_1−x_Na_x_Ta_1−y_Nb_y_O_3_crystals.

Composition of the KNTN crystal	K/Na in the melt		Ta/Nb in the melt	
K_0.30_Na_0.70_Ta_0.37_Nb_0.63_O_3_	75:25		10:90	
K_0.36_Na_0.64_Ta_0.40_Nb_0.60_O_3_	75:25		15:85	
K_0.39_Na_0.61_Ta_0.42_Nb_0.58_O_3_	75:25		18:82	
Segregation of K	Segregation of Na	Segregation of Ta	Segregation of Nb	*T*_g_(°C)
0.400	2.800	3.700	0.700	1193.0
0.480	2.560	2.667	0.706	1245.0
0.520	2.440	2.333	0.707	1277.5

**Table 2:** Lattice parameters of the KNTN crystals.

Crystal	*a*(Å)	*b*(Å)	*c*(Å)
K_0.30_Na_0.70_Ta_0.37_Nb_0.63_O_3_	5.6061	3.9385	5.5876
K_0.36_Na_0.64_Ta_0.40_Nb_0.60_O_3_	5.6083	3.9433	5.5828
K_0.39_Na_0.61_Ta_0.42_Nb_0.58_O_3_	5.6290	3.9449	5.5828
K_0.3_Na_0.7_NbO_3_^40^	5.64304	3.93187	5.61260

^a^The lattice parameters corresponding to a symmetric orthorhombic cell were converted into the lattice parameters for a pseudo-monoclinic cell (*a*′ = *c*′, *b*, and *β*) using the following formula *a* = 2*d* sin (*β*/2), *c* = 2*a*′ sin (*β*/2)^41^.

**Table 3:** The *T*_O-T_, *T*_C_, dielectric constant (*ε*_r_), and dielectric loss (tan*δ*) values of the KNTN single crystal at a frequency of 1 kHz.

Crystal	*T*_O-T_(°C)	*T*_C_(^o^C)	*ε*_r_ at *T*_room_	tan*δ* at *T*_room_ (%)
K_0.30_Na_0.70_Ta_0.37_Nb_0.63_O_3_	84	228	369	0.7
K_0.36_Na_0.64_Ta_0.40_Nb_0.60_O_3_	58	209	609	0.7
K_0.39_Na_0.61_Ta_0.42_Nb_0.58_O_3_	39	161	670	0.6
K_0.334_Na_0.666_NbO_3_^26^	174	395	68	0.4
K_0.5_Na_0.5_NbO_3_ 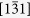 29	192	410	1015	1

**Table 4:** Coercive field (*E*_c_), remanent polarization (*P*_r_), and spontaneous polarization (*P*_s_) values of the K_1−x_Na_x_Ta_1−y_Nb_y_O_3_ single crystals.

	*E*_c_ (kV/cm)	*P*_r_ (μC/cm^2^)	*P*_s_(μC/cm^2^)
K_0.30_Na_0.70_Ta_0.37_Nb_0.63_O_3_	6.52	6.11	9.14
K_0.36_Na_0.64_Ta_0.40_Nb_0.60_O_3_	5.05	5.37	10.34
K_0.39_Na_0.61_Ta_0.42_Nb_0.58_O_3_	6.73	6.59	9.86
K_0.334_Na_0.666_NbO_3_^26^	11.59	~12	~12
K_0.455_Na_0.545_NbO_3_^26^	11.11	~12	~12

**Table 5:**
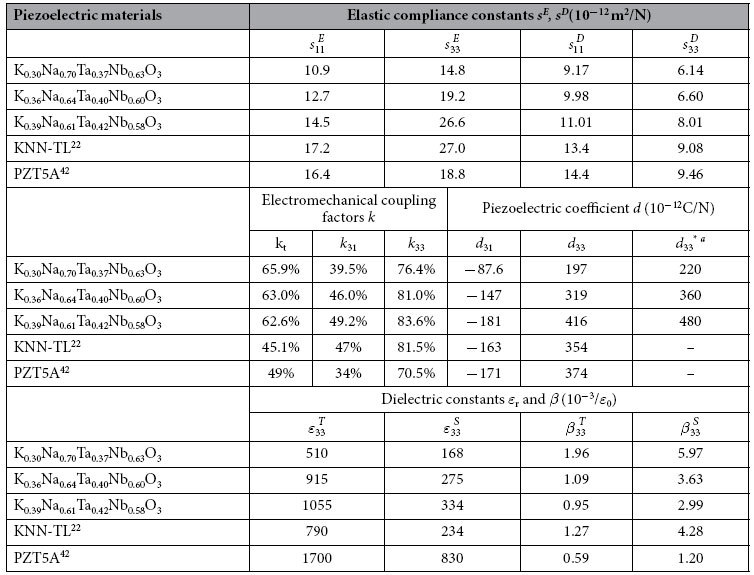
Piezoelectric parameters of the as-grown KNTN single crystals poled along [001]_c_ and having tetragonal 4*mm* symmetry.

^a^Measured using a piezo-*d*_33_ meter.

**Table 6:** Internal energies of the K_1−x_Na_x_Ta_1−y_Nb_y_O_3_ crystals.

K_1−x_Na_x_Ta_1−y_Nb_y_O_3_	*U*_o_(eV/Å^3^)	*U*_t_ (eV/Å^3^)	*ΔU* = *U*_t_ * − U*_o_ (eV/Å^3^)
K_0.30_Na_0.70_Ta_0.37_Nb_0.63_O_3_	50.7596	53.7836	3.0241
K_0.36_Na_0.64_Ta_0.40_Nb_0.60_O_3_	49.6940	52.4140	2.7200
K_0.39_Na_0.61_Ta_0.42_Nb_0.58_O_3_	49.0279	51.5945	2.5666
